# Identification of Eleven Novel *BRCA* Mutations in Tunisia: Impact on the Clinical Management of *BRCA* Related Cancers

**DOI:** 10.3389/fonc.2021.674965

**Published:** 2021-08-20

**Authors:** Yosr Hamdi, Najah Mighri, Maroua Boujemaa, Nesrine Mejri, Sonia Ben Nasr, Mariem Ben Rekaya, Olfa Messaoud, Hanen Bouaziz, Yosra Berrazega, Haifa Rachdi, Olfa Jaidane, Nouha Daoud, Aref Zribi, Jihene Ayari, Houda El Benna, Soumaya Labidi, Jamel Ben Hassouna, Abderazzek Haddaoui, Khaled Rahal, Farouk Benna, Ridha Mrad, Slim Ben Ahmed, Hamouda Boussen, Samir Boubaker, Sonia Abdelhak

**Affiliations:** ^1^Laboratory of Biomedical Genomics and Oncogenetics, LR20IPT05, Institut Pasteur de Tunis, University of Tunis El Manar, Tunis, Tunisia; ^2^Laboratory of Human and Experimental Pathology, Institut Pasteur de Tunis, Tunis, Tunisia; ^3^Medical Oncology Department, Abderrahman Mami Hospital, Faculty of Medicine Tunis, University Tunis El Manar, Tunis, Tunisia; ^4^Department of Medical Oncology, Military Hospital of Tunis, Tunis, Tunisia; ^5^UR17ES15, Oncotheranostic Biomarkers, Faculty of Medicine of Tunis, University Tunis El Manar, Tunis, Tunisia; ^6^Surgical Oncology Department, Salah Azaiez Institute of Cancer, Tunis, Tunisia; ^7^Department of Radiation Oncology, University of Tunis, Tunis, Tunisia; ^8^Department of Human Genetics, Charles Nicolle Hospital, Tunis, Tunisia; ^9^Faculty of Medicine of Sousse Department of Medical Oncology Farhat Hached University Hospital University of Sousse, Sousse, Tunisia

**Keywords:** *BRCA* cancers, genetic testing, novel *BRCA* mutations, clinicopathological signatures, precision medicine

## Abstract

**Background:**

Breast cancer is the world’s most common cancer among women. It is becoming an increasingly urgent problem in low- and middle-income countries (LMICs) where a large fraction of women is diagnosed with advanced-stage disease and have no access to treatment or basic palliative care. About 5-10% of all breast cancers can be attributed to hereditary genetic components and up to 25% of familial cases are due to mutations in *BRCA1*/2 genes. Since their discovery in 1994 and 1995, as few as 18 mutations have been identified in *BRCA* genes in the Tunisian population. The aim of this study is to identify additional *BRCA* mutations, to estimate their contribution to the hereditary breast and ovarian cancers in Tunisia and to investigate the clinicopathological signatures associated with *BRCA* mutations.

**Methods:**

A total of 354 patients diagnosed with breast and ovarian cancers, including 5 male breast cancer cases, have been investigated for *BRCA1/2* mutations using traditional and/or next generation sequencing technologies. Clinicopathological signatures associated with *BRCA* mutations have also been investigated.

**Results:**

In the current study, 16 distinct mutations were detected: 10 in *BRCA1* and 6 in *BRCA2*, of which 11 are described for the first time in Tunisia including 3 variations that have not been reported previously in public databases namely *BRCA1*_c.915T>A; *BRCA2*_c.-227-?_7805+? and *BRCA2*_c.249delG. Early age at onset, family history of ovarian cancer and high tumor grade were significantly associated with *BRCA* status. *BRCA1* carriers were more likely to be triple negative breast cancer compared to *BRCA2* carriers. A relatively high frequency of contralateral breast cancer and ovarian cancer occurrence was observed among *BRCA* carriers and was more frequent in patients carrying *BRCA1* mutations.

**Conclusion:**

Our study provides new insights into breast and ovarian cancer genetic landscape in the under-represented North African populations. The prevalence assessment of novel and recurrent *BRCA1/2* pathogenic mutations will enhance the use of personalized treatment and precise screening strategies by both affected and unaffected North African cancer cases.

## Introduction

Breast cancer is the most common malignancy among women worldwide (1). Incidence and mortality rates of breast cancer differ between populations (1). In Tunisia, it remains the most common cancer among females and represents the first leading cause of cancer mortality among women. The mean age at diagnosis of Tunisian breast cancer cases is around 50 years old, a decade younger than Western countries (2, 3).

*BRCA1* and *BRCA2* are the most prominent breast cancer susceptibility genes that convey high risk of breast and ovarian cancers (4). Since their discovery, a wide range of mutational spectrum have been described for both genes. So far, more than 1800 distinct *BRCA1* and 2000 *BRCA2* mutations have been reported in the Breast Cancer Information Core (BIC) database. These mutations explain around 20-30% of breast cancer genetic component and seem to be associated with different other cancers such as prostate, pancreatic, endometrial and melanoma (5). The identification of novel *BRCA1/2* mutations has important clinical implications. Indeed, unaffected *BRCA* mutation carriers have various preventive options including extensive and regular surveillance, chemoprevention, and risk-reducing surgery (6–8), while, affected cases carrying *BRCA* mutations could benefit from personalized therapeutic options such as platinum-based chemotherapy and poly (ADP-ribose) polymerase (PARP) inhibitors (9, 10). However, a full *BRCA1* and *BRCA2* gene screening remains a labor and time-consuming challenge due to the large gene size, diverse mutations, or variants of unknown significance (VUS) and complexity of large genomic rearrangements (LRs) and copy number variations (CNVs) requiring special technical approaches. Recent advances in high throughput sequencing technologies including Target panels and whole exome sequencing (WES) allowed rapid, sensitive, and cost-effective screening of the large *BRCA* genes. In addition, the decreased cost of genotyping and sequencing offered affordable targeted testing options.

Sequencing thousands of cancer samples showed that the frequency of germline mutations in *BRCA* genes varies widely among populations. Some mutations are shared between different populations and others are ethnic specific (11, 12). Indeed, in certain countries and ethnic communities, the *BRCA* mutation spectrum is limited to a few founder mutations (13, 14). This is mainly observed in geographically, culturally, or religiously isolated populations and in countries with high rates of consanguinity and endogamy that undergo rapid expansion from a limited number of ancestors. Consequently, some alleles become more frequent which explain the high frequency of some founder mutations in these populations. The founder effect may, therefore, influence mutation prevalence and gene penetrance. Since cancer risk is a function of mutation prevalence and penetrance that seems to vary by ethnicity, investigating the prevalence, the frequency, and the penetrance of novel *BRCA* mutation in different populations will bring new insights on cancer risk and etiology.

In Tunisia, previous studies on *BRCA* genes have focused only on breast cancer patients. In some studies, the genetic investigation concerned all the coding regions of *BRCA1* and/or *BRCA2* genes and in others only hotspot exons have been investigated. These reports have revealed a total of only 18 distinct mutations of which 12 are localized within *BRCA1* gene including 2 large rearrangements encompassing exons 5 and 20. Among the identified mutations c.211dupA, c.5266dupC in *BRCA1* and c.1310_1313delAAGA in *BRCA2* were the most recurrent mutations encountered among the hereditary breast cancer cases (11, 15–23). Despite these efforts, the mutational spectrum of *BRCA1/2* genes is still not well established. The main goal of the present study was to identify additional novel *BRCA* mutations and to investigate the contribution of these mutations to the missing heredity of breast and ovarian cancers. We also aimed to compare breast cancer clinicopathological characteristics in BRCA+ vs *BRCA*- Tunisian breast cancer cases.

## Materials And Methods

### Patients

**Figure 1:**
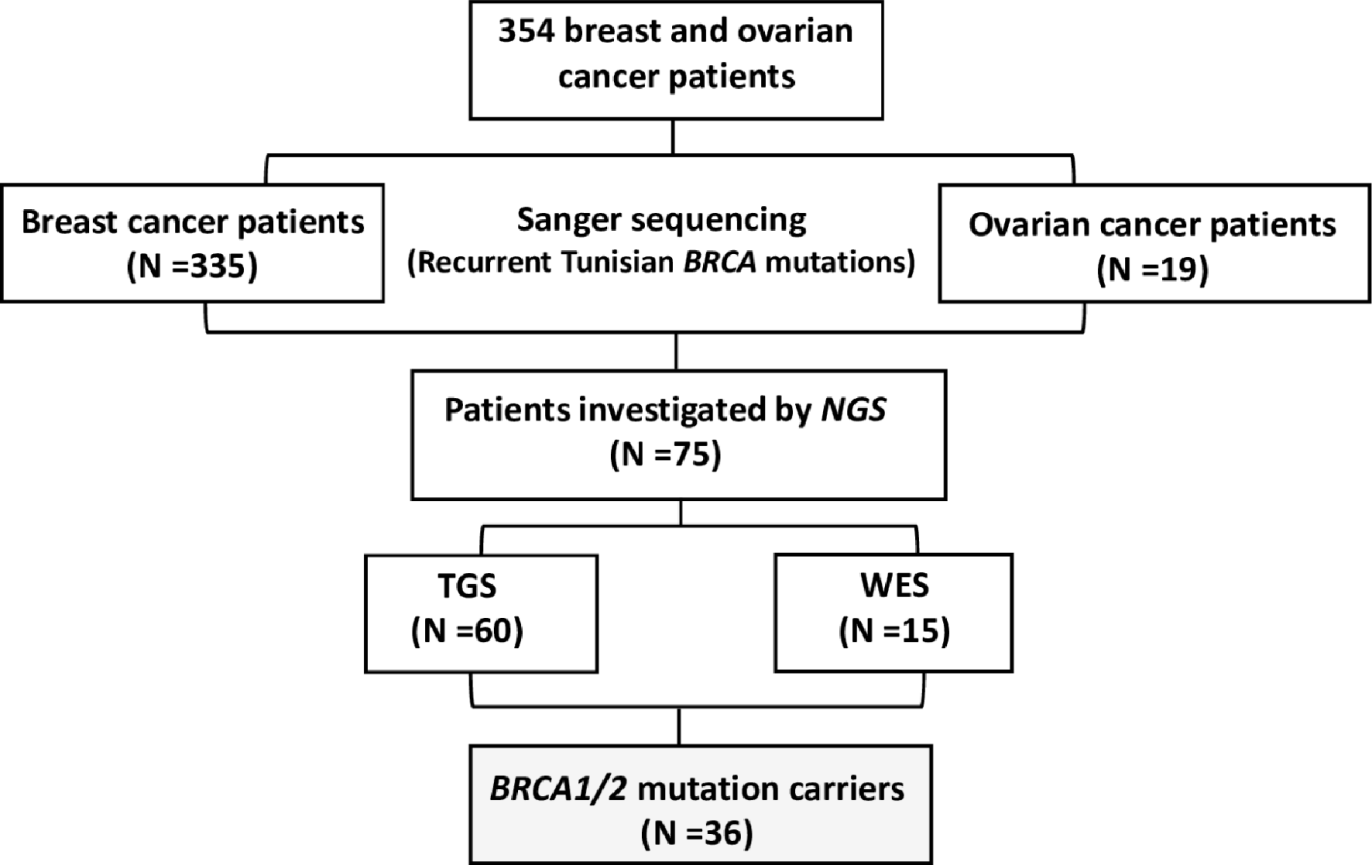
Study flowchart.

A total of 354 breast and ovarian cancer patients (335 breast cancer patients and 19 ovarian cancer patients) were included in this study referred from different medical oncology departments in Tunisia including those of Abderrahman Mami Hospital, Military Hospital of Tunis, Salah Azaiez Institute of Cancer and Farhat Hachad University Hospital University of Sousse. Written informed consents were obtained from all participants. The study has been conducted according to the Declaration of Helsinki Principles and ethical approval was obtained from the biomedical ethics committee of Institut Pasteur de Tunis (2017/16/E/Hôpital A-M). Clinico-pathological characteristics and follow-up data were collected from patients’ medical records. Probands were selected based on the following selection criteria (1): Presence of at least 3 related first or second-degree breast cancer cases at any age (2), Young cancer patients aged less than 35 years (3), Presence of at least two cases of breast or ovarian cancer, regardless of age, and at least one case of pancreatic cancer or prostate cancer in a related first- or second-degree patient (4), one case with triple negative breast cancer (TNBC) at an age ≤40 years (5), one breast cancer and one ovarian cancer cases diagnosed at first or second degree relatives at any age. A study flowchart is illustrated in **Figure 1**.

### DNA Isolation

Total genomic DNA was isolated from peripheral blood using DNeasy blood DNA extraction Kit (Qiagen) according to the manufacturer’s instructions. DNA purity and concentration were measured using a NanoDrop™ spectrophotometer.

### Screening for Recurrent Mutations in *BRCA1* and *BRCA2* Genes Using Sanger Sequencing

Before performing Next Generation Sequencing (NGS) analysis, the studied cohort was screened for at least one of the recurrent *BRCA1/2* mutations previously reported in the Tunisian population, namely exon5-c.211dupA (rs397508938), exon11-c.798_799delTT (rs80357724), exon11-c.2551delG (rs397508977), exon11-c.3331_3334delCAAG (rs80357701) and exon20-c.5266dupC (rs80357906) of the *BRCA1* gene and exon10-c.1310_1313 delAAGA (rs80359277), exon16-c.7654dupA (rs879255463) in *BRCA2* gene respectively. The reference sequences used were NM_007294.3 for *BRCA1* and NM_000059.3 for *BRCA2*.

PCR reactions were performed on genomic DNA (gDNA), following standard protocols. Sanger sequencing has been performed using an automated sequencer (ABI 3500; Applied Biosystems, Foster City, CA) and a cycle sequencing reaction kit (Bigdye Terminator v3.1 kit, Applied Biosystems). The data were analyzed using BioEdit software version 7.2.5.

Sanger sequencing technique was then used to validate the identified mutations resulting from NGS.

NGS was performed on 75 breast and ovarian cancer cases. Targeted *BRCA1/2* sequencing and whole exome sequencing were performed on 60 and 15 patients respectively.

### Targeted Gene Sequencing

Targeted gene sequencing was performed on *BRCA1/2* for 60 breast and ovarian cancer patients with strong family history. All targeted coding exons and exon–intron boundaries of *BRCA1/2* genes were amplified with 253 pooled primer pairs. After the targeted amplification and construction of a library through QIAGEN Library Kit v2.0, the libraries were pooled prior to emulsion PCR and bead enrichment steps that were carried out using an automated protocol on the GeneRead QIAcube (QIAGEN, Hilden, Germany) using the GeneRead Clonal Amp Q Kit (QIAGEN, Hilden, Germany), according to the manufacturer’s protocol. Following bead enrichment, the pooled libraries were sequenced using the GeneReader platform (QIAGEN, Hilden, Germany).

### Whole Exome Sequencing

WES was performed for 15 breast cancer Tunisian patients. Samples were prepared according to Agilent’s SureSelect Protocol Version 1.2 and enrichment was carried out according to Agilent SureSelect protocols. Enriched samples were sequenced on the Illumina HiSeq2000 platform using TruSeq v3 chemistry with paired-end (2 × 100). Exome DNA sequences were mapped to their location in the build of the human genome (hg19/b37) using the Burrows–Wheeler Aligner (BWA) package. The subsequent SAM files were converted to BAM files using Samtools. Duplicate reads were removed using Picard. GATK was then used to recalibrate the base quality scores as well as for SNP and short INDEL calling. Annotation and prioritization of potential disease-causing variants were performed using VarAFT (Variant Annotation and Filtering Tool) (http://varaf t.eu). To annotate variants, VarAFT uses ANNOVAR, a command line tool. INDELs and SNPs annotated were filtered according to several criteria (1): considering breast cancer as autosomal dominant disease and removing variants that were found in a homozygous state (2), variants identified as intronic, intergenic, and non-coding or synonymous were discarded (3), assuming that causal variants are rare, we removed all variants with an allele frequency > 1% either in ExAC (24), 1000 genomes (25) or ESP6500 (http://evs.gs.washington.edu/EVS/) (4), Using different *in silico* prediction tools, the functional impact of all identified variants has been assessed. Based on this assessment, Benign and tolerated variants were removed. Finally, significant candidate variants were obtained after filtering against their phenotypic relevance.

### Clinico-Pathological Features of *BRCA1* and *BRCA2* Carriers

Clinical and pathological features of *BRCA*+ vs *BRCA*- patients as well as *BRCA1* vs *BRCA2* carriers were compared and evaluated. Statistical analysis was performed using SPSS software (version 23). Quantitative variables with normal distribution were analyzed by Student’s t test. Comparison of qualitative data was performed using Chi-square test. Fisher’s exact test was used for the study of small sample size. Correlation is considered statistically significant between two variables if the *P* value is less than or equal to 0.05.

## Results

### Epidemiological and Clinico-Pathological Features of Investigated Breast and Ovarian Cancer Patients

A family history of breast and ovarian cancer was present in 35.24% and 11.14% of patients respectively. In addition, 2.68% of patients presented both breast and ovarian cancers. Consanguineous families represent 35.31% of the studied patients. Mean age at menarche was 12.81 years. Mean age at first pregnancy was 26.62 years. Oral contraception was reported by 47.31% of patients, 25.99% of patients have never breastfed and 31.85% were premenopausal.

The mean age at diagnosis of breast cancer was 43.10 years and 31.94% of patients were ≤35 years. Among investigated patients1.49% were male breast cancer (MBC) cases. Inflammatory breast cancer (IBC) (T4d) was seen in 8.65% of patients. Invasive ductal carcinoma (IDC) was the most frequent (90.04%) while infiltrating lobular carcinoma (ILC) was observed in only 3.94% of cases. Scarff-Bloom-Richardson (SBR) grade III was the most common (47.71%). Mean Tumor size was 33.62mm. Patients with positive lymph node disease represented 53.37% of our cohort, 88.67% of patients had Ki-67>14%. Luminal B tumors were the most common (56.88%) followed by triple negative breast cancer (TNBC) (23.85%), Her2+ (11.93%) and luminal A (7.34%). Distant metastases were observed in 26.34% of patients.

For ovarian cancer cases, the mean age at diagnosis was 52.62 years and the majority with serous ovarian carcinoma.

### Genetic Analysis

Genetic analysis results showed that 36 out of 354 tested breast and ovarian cancer patients were *BRCA1/2* mutation carriers (31 breast cancer cases and 5 ovarian cancer patients), including 21 patients with *BRCA1* mutation and 15 patients carrying *BRCA2* mutation. A total of 16 mutations have been identified including 11 short indels, 4 single nucleotide variations (3 nonsense & 1 splicing) and 1 large rearrangement.

### Identified *BRCA1/2* Pathogenic Mutations in Breast Cancer Cases

Within the studied breast cancer cohort, 13 pathogenic mutations have been identified: 8 in *BRCA1* and 5 in *BRCA2* genes. Among the identified mutations, 9 are described for the first time in Tunisian population (6 in *BRCA1* and 3 in *BRCA2*) **(****Table 1****)**.

**Table 1:** Mutations in the *BRCA1/2* genes identified in breast cancer and ovarian cancer patients by Sanger and next generation sequencing technologies.

Gene	Exon	Coding change	Protein variation	dbSNP rs ID	Number of families carrying mutations	Number of patients carrying mutations	Screening method
***BRCA1***	2	c.19_47del	p.Arg7fs*24	rs80359871	1	1 (BC)	NGS
	5	c.211dupA	p.Arg71fs*10	rs397508938	5	6 (BC)	Sanger sequencing
	10	c.668dupA	p.Ala224Glyfs*4	rs80357537	1	1 (BC)	NGS
	11	c.915T>A	Cys305*	–	1	1 (OC)	Sanger Sequencing
	11	c.1612C>T	p.Gln538*	rs80356893	3	4 (2 BC, 2 OC)	NGS
	11	c.2418dupA	p.Ala807Serfs*3	rs886040036	1	1 (BC)	NGS
	11	c.2433delC	p.Lys812fs*3	rs80357524	1	2 (BC)	NGS
	11	c.3049G>T	Glu1017*	rs80357004	1	1 (OC)	NGS
	17	c.5030_5033delCTAA	p.Thr1677fs*2	rs80357580	1	1 (BC)	NGS
	20	c.5266dupC	p.Gln1756Profs*74	rs80357906	2	3 (BC)	Sanger sequencing
***BRCA2***	3	c.249delG	p.Glu83Aspfs	–	1	1 (OC)	NGS
	8	c.632-1G>A	–	rs81002820	5	6 (BC)	NGS
	10	c.1310_1313delAAGA	p.Lys437fs*22	rs80359277	9	10 (9 BC, 1 MBC)	NGS/Sanger sequencing
	10	c.1389_1390delAG	p.Val464fs*3	rs80359283	1	1 (MBC)	NGS
	16	c.7654dupA	p.Ile2552Asnfs*2	rs879255463	1	2 (BC)	Sanger sequencing
	1-16	c.-227-?_7805+?	–	–	1	1 (BC)	NGS

BC, breast cancer; MBC, Male Breast Cancer; OC, ovarian cancer; NGS, Next Generation Sequencing.

The * symbol design the codon stop/frameshift mutation (fs).

Considering the *BRCA1* gene, 6 patients belonging to 5 unrelated families were carriers of the recurrent c.211dupA mutation. Three patients belonging to 2 unrelated families were positive for c.5266dupC mutation. The missense c.1612C>T mutation has been identified in 2 related patients. c.19_47del, c.668dupA, c.2418dupA and c.5030_5033delCTAA mutations have been identified each in one patient. c.2433delC has been identified among 2 related patients. Except c.211dupA and c.5266dupC mutations, all remaining *BRCA1* mutations are reported for the first time in the Tunisian population.

In the *BRCA2* gene, 3 frameshift mutations as well as 1 splicing and 1 large rearrangement mutation were detected. Our results revealed 6 patients belonging to 5 unrelated families that are double heterozygous for *BRCA2* gene. Indeed, these families were carrying two mutations classified as pathogenic in the ClinVar database namely c.632-1G>A and c.1310_1313delAAGA. Four additional patients carrying only the c.1310_1313delAAGA mutation have been identified including one male breast cancer. The c.7654dupA mutation was identified in 2 related patients with a strong family history of hereditary breast and ovarian cancer. The c.1389_1390delAG mutation has been identified in 1 additional male breast cancer case. Similarly, a large rearrangement mutation of *BRCA2* gene (Del exons 1-16) has been identified in one patient. Among the identified *BRCA2* mutations, *BRCA2*-Del exons 1-16 mutation is novel and was not described in public databases. c.632-1G>A and c.1389_1390delAG are described for the first time in the Tunisian population.

### *BRCA1/2* Pathogenic Mutations Identified in Ovarian Cancer Cases

A total of 19 ovarian cancer patients were screened for *BRCA* pathogenic mutations using Sanger and/or NGS. Four distinct deleterious mutations were identified: 3 mutations in *BRCA1* gene (c.915T>A, c.1612C>T and c.3049G>T) and one *BRCA2* mutation (c.249delG).

**Figure 2:**
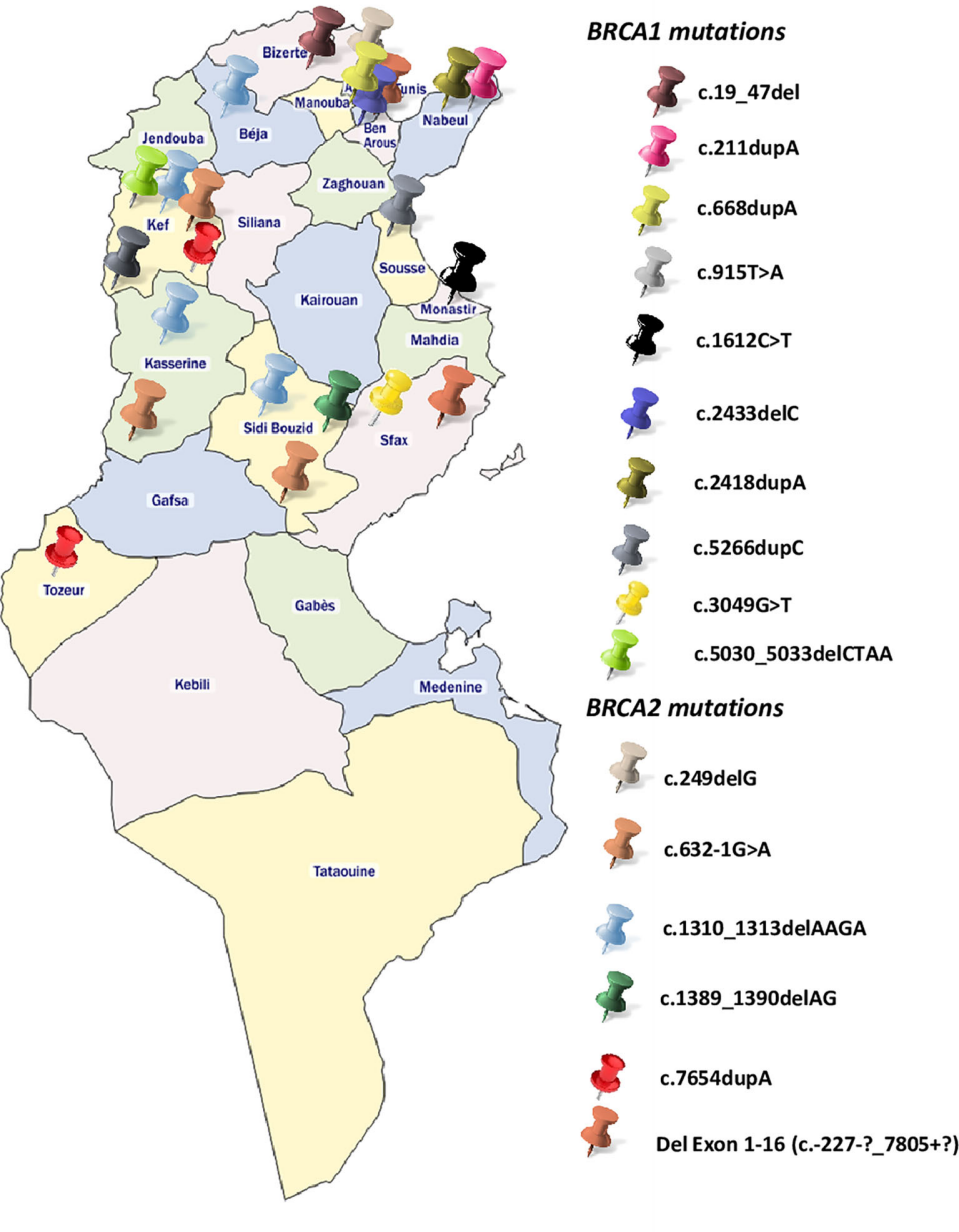
Geographical distribution of the identified *BRCA1* and *BRCA2* mutations.

**Figure 3:**
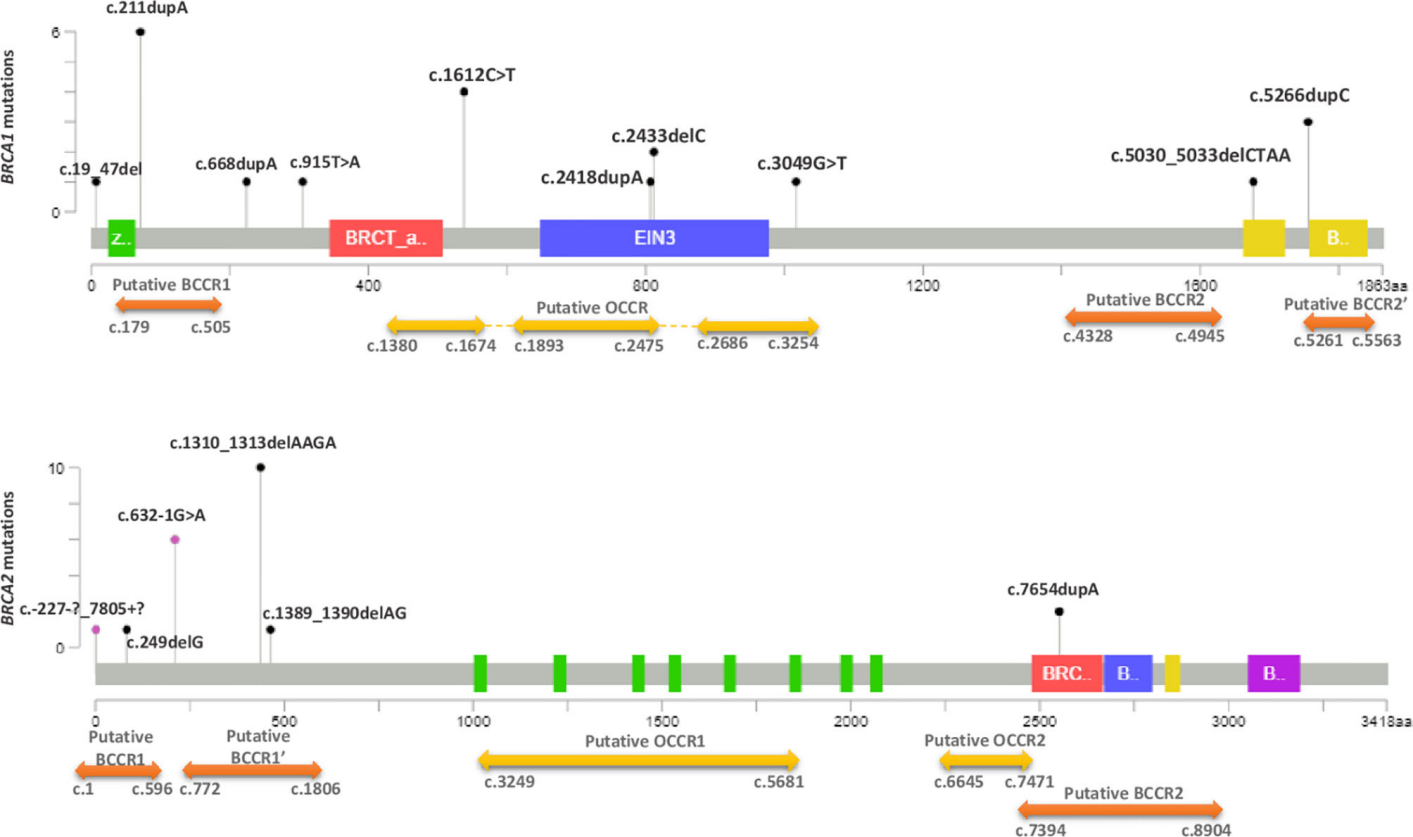
Distribution of *BRCA1* and *BRCA2* mutations identified in hereditary breast and ovarian cancer cases. The Length of mutation indicator reflects the number of observed carriers. The diagrams linearly represent BRCA1/2 protein domains (x-axis). BRCA1 domains: Zinc/Ring finger (green); BRCT_assoc: serine-rich domain associated with BRCT (red); Ethylene insensitive 3 (blue); BRCA1 C terminus domain (yellow). BRCA2 domains: BRC repeats (green); BRCA-2_helical (red); oligonucleotide/oligosaccharide-binding, domain 1 (blue); tower domain (yellow) and oligonucleotide/oligosaccharide-binding, domain 3 (purple). Black mutation indicators depict truncating mutations and purple indicators represent the other types of mutations (splicing, LR). Cluster regions (breast cancer cluster regions (BCCRs) (orange) and ovarian cancer cluster regions (OCCRs) (yellow) were assigned according to the study of Rebbeck et al. (26).

*BRCA1*-c.915T>A and *BRCA2*-c.249delG mutations are novel and not described in public databases. The other identified mutations are described for the first time in the Tunisian population. c.1612C>T mutation was identified among 2 patients. This same mutation was also identified in 2 related breast cancer patients. Screening for additional carriers of the identified mutations, based on their geographic origin, was performed using Sanger sequencing. Consequently, the geographic origin of the identified *BRCA1/2* mutations has been clearly established (**Figure 2**). We have also illustrated the distribution of *BRCA* mutations identified in hereditary breast and ovarian cases on *BRCA1* and *BRCA2* genes (**Figure 3**). Breast cancer cluster regions (BCCRs) and ovarian cancer cluster regions (OCCRs) were assigned in **Figure 3** according to the study of Rebbeck et al., 2015 (26). Among *BRCA* mutations identified in breast cancer patients, *BRCA1_c*.211dupA, *BRCA1*_c.5266dupC, *BRCA2*_c.-227-?_7805+?, *BRCA2*_1310_1313delAAGA, *BRCA2*_c.1389_1390delAG and *BRCA2*_c.7654dupA occurred in BCCRs. Considering *BRCA* mutations identified in ovarian cancer patients *BRCA1*_c.1612C>T and *BRCA1*_3049G>T arose in OCCRs.

### Polymorphisms and Variant of Unknown Significance Identified in *BRCA1* and *BRCA2* Genes

In addition to the pathogenic mutations that have been identified in *BRCA* genes, several SNPs and variants of unknown significance (VUS) have been observed **(****Supplementary Table 1****)**. Among the 101 identified variants, 45.54% were coding, 50.49% were intronic and 3.96% were localized within regulatory regions. The majority of variations were classified as benign or likely benign in the ClinVar database (91.08%) and five intronic variations were not reported. One patient carried a VUS rs397507308 in *BRCA2* and 3 other patients carried 2 intronic variations (rs276174878 and rs276174816) that have conflicting interpretations of pathogenicity. Another patient diagnosed with early onset bilateral breast cancer had an in-frame variant with conflicting interpretations of pathogenicity rs80358343 (c.5017_5019delCAC) in the *BRCA1* gene.

### Clinico-Pathological Features of *BRCA* Carriers Among Breast Cancer Cohort

Clinico-pathological characteristics of breast cancer cases carrying *BRCA1* and *BRCA2* mutations are described in **Tables 2**, **3** respectively. We investigated these clinico-pathological features in *BRCA1/2* mutation carriers vs *BRCA negative patients* (**Table 4**) and between *BRCA1* and *BRCA2* mutation carriers (**Table 5**), as well.

**Table 2:** Clinicopathological features of *BRCA1* carriers.

Mutation	Carrier ID	Pathology	Age at diagnosis (years)	Family History BC/OC	Family history of other cancers	Histological subtype	SBR grade	ER status	PR status	HER2 status	Ki67- index (%)	Nodal status	Tumor size (mm)	Follow-up
**c.19_47del**	BC320-1	BC	35	3 BC	1 gastric, 2 lung, 1 esophageal	IDC	III	ER+	PR-	HER2+	70%	N+	25	Bone and lung metastases at 37 years old.
**c.211dupA**	BC9-1	BC/OC/CBC	42	1 BC/OC 1 OC	1 Cervical cancer	IDC	NA	NA	NA	NA	NA	NA	NA	OC at 57 years old CBC at 63 years old Died at 67 years old
	BC49-1	BC/CBC	29	2 BC	Leukemia, Prostate, Colon, Gynecological cancer, Larynx	IDC	III	ER-	PR-	HER2-	NA	N+	35	Spontaneous pregnancy 6 months after the end of CT CBC at 32 years old (ER+, PR-, HER2-)
	BC49-2	BC	37	2 BC		IDC	III	ER +	PR +	HER2-	40	N+	15	Patient in complete remission, under regular surveillance.
	BC199-1	BC	58	1 BC 1 OC	1 Endometrium	IDC	III	ER -	PR -	HER2-	2	N+	40	Bone and Lung metastases at initial diagnosis Disease progression, cerebral metastases Died at 59 years old
	BC204	BC/OC	28	2 BC 2 OC	1 Thyroid	IDC	NA	ER -	PR -	HER2-	20	N-	30	OC at 41 years old. Patient in complete remission, under regular surveillance.
	PEC50-1	BC	38	2 BC	2 Lung, 1 Pancreatic	IDC	II	ER -	PR-	HER2-	NA	NA	30	NA
**c.668dupA**	BC420	BC	64	1 BC	1 Colorectal,1 tongue cancer	NA	NA	NA	NA	NA	30	NA	NA	NA
**c.1612C>T**	BC276-1	BC	25	4 BC 1 BOC 1 OC	1 Lung, 1 head and neck	IDC	II	ER -	PR-	HER2-	NA	N+	22	Disease progression, multiple metastases
	BC276-3	BC/OC	ND	1 BC 1 OC	1 Lung	NA	NA	NA	NA	NA	80	NA	NA	Discovery of ovarian involvement during a preoperative examination for a prophylactic oophorectomy
**c.2418dupA**	BC93	IBC	34	None	1 Lung, 1 pancreatic	IDC	II	ER -	PR -	HER2-	NA	NA	NA	Bone metastases Died at 36 years old due to disease progression
**c.2433delC**	BC178-1	BC/CBC/Endometrial cancer	42	2 BC 1 OC	2 Lung, 1 colorectal, 1 bladder	IDC	III	NA	NA	NA	30	N+	25	CBC at 45 years old; Endometrial cancer at 55 years old with peritoneal metastases
	BC178-2	BC/CBC	45			IDC	II	ER -	PR -	HER2-	NA	N-	13	CBC at 52 years old
**c.5030_5033delCTAA**	BC70	BC/OC	47	3 BC 1 OC	1 Lung	Polymorphic IDC	III	ER -	PR -	HER2-	65	N-	30	Complete remission Died at 58 years old
**c.5266dupC**	BC81-1	BC/(CBC: PABC)	27	4 BC	1 Pancreatic 1 colorectal cancer	IDC	III	ER -	PR -	HER2-	NA	N-	24	PABC at 32 years
	BC81-6	BC	36			IDC	III	ER -	PR -	HER2+	NA	NA	20	NA
	BC314	BC	29	6 BC	1 Prostate	IDC	III	ER +	PR +	HER2-	40	NA	NA	NA

BC, breast cancer; OC, ovarian cancer; CBC, contralateral breast cancer; IDC, invasive ductal carcinoma; PABC, Pregnancy associated breast cancer; NA, non available; PR, Progesterone receptor; ER, Estrogen receptor.

**Table 3:** Clinico-pathological features of *BRCA2* carriers.

Mutation	Carrier ID	Pathology	Age at diagnosis (years)	Family History BC/OC	Family history of other cancers	Histological subtype	SBR grade	ER status	PR status	HER2 status	Nodal status	Tumor size (mm)	Ki67- index (%)	Follow-up
**DH (c.632-1G>A, c.1310_1313delAAGA**	BC6-1	BC	40	4 BC	1 Throat cancer	IDC	NA	NA	NA	NA	NA	NA	NA	Died at 44 years old
	BC17-1	BBC	36	9 BC	1 Gastric cancer	IDC	III	ER+	PR+	NA	N+	25	NA	Esophageal Carcinoma at 48 years old. Disease progression, laterocervical, bone and liver metastases. Died at 50 years old
					1 Kidney cancer									
	BC17-2	BC (PABC)/CBC	25	9 BC	1 Gastric cancer	IDC	II	ER +	PR +	HER2-	N+	55	20	CBC Bone metastases, Unplanned pregnancy during BC treatment Lung metastases at 27 years old
					1 Kidney cancer									
	BC39	BC	27	None	None	IDC	III	ER +	PR +	HER2-	NA	NA	NA	Bone and liver metastases at initial diagnosis Disease progression, patient died at 35 years old
	BC95	BC	32	None	1 Colorectal cancer	IDC	I	ER+	PR+	HER2-	N-	7	50	Patient in complete remission, under regular surveillance
	BC225-1	BC	50	5 BC	1 cerebral cancer	IDC	III	ER+	PR+	HER2-	N+	20	60	Under regular surveillance
					2 esophageal									
**c.1310_1313delAAGA**	BC245	IBC (PABC) CBC	36	2 BC	None	IDC	II	ER+	PR+	HER2-	N+	NA	80	CBC at 37 years old
	PEC009	BC	33	1 BC	None	IDC	II	ER+	PR+	HER2-	N+	NA	25	Under regular surveillance
	PEC0035	MBC	43	2 MBC	None	IDC	III	ER+	PR+	HER2-	N+	7	30	Under regular surveillance
				1 BC										
	BC354-1	BC	37	2 BC	1 pancreatic	ILC	I	ER+	PR+	HER2-	N+	25	15	NA
				1 IBC	1 Lung									
				1 MBC										
**c.1389_1390delAG**	PEC0056	MBC	59	1 BC	1 bladder	IDC	III	ER+	PR -	HER2-	N+	54	30	Under regular surveillance
**c.7654dupA**	BC231-1	BC/OC	47	8 BC	2 Gastric,	IDC	III	ER+	PR +	HER2-	N-	55	NA	OC at 51 years old
				1 BOC	1 prostate,									
					1 hepatic cancers									
	BC231-2	BC	34			IDC	III	ER+	PR +	HER2-	N+	16	20	Under regular surveillance
**c.-227-?_7805+?**	BC287	IBC (PABC)	36	3 BC 1 BBC 1 BOC 1MBC/prostate cancer	1 Larynx	IDC	III	ER+	PR +	HER2-	NA	NA	40	Contralateral prophylactic mastectomy Under regular surveillance
				1 IBC										

BC, breast cancer; OC, ovarian cancer; CBC, contralateral breast cancer; IDC, invasive ductal carcinoma; PABC, Pregnancy associated breast cancer; NA, non available; PR, Progesterone receptor; ER, Estrogen receptor.

**Table 4:** Epidemiological and clinico-pathological characteristics of patients carrying or not *BRCA1/2* mutations.

Variables	*BRCA1/2+*N=31	*BRCAx*N=52	*P* value
**Mean age at diagnosis (years)**	38.37	43.14	0.049
**Early age at onset (≤35 years)**			
**Yes**	12/30 (40.0%)	11/50 (22%)	0.085
**No**	18/30 (60.0%)	39/50 (78%)	
**Family history of breast cancer**			
**Yes**	28/31 (90.32%)	42/52 (80.77%)	0.353
**No**	3/31 (9.68%)	10/52 (19.23%)	
**Family history of ovarian cancer**			
**Yes**	11/31 (35.48%)	5/51 (9.80%)	0.004
**No**	20/31 (64.52%)	46/51 (90.20%)	
**Personal history of cancers**			
**Yes**	5/31 (16.13%)	4/51 (7.84%)	0.288
**No**	26/31 (83.87%)	47/51 (92.16%)	
**Consanguinity**			
**Yes**	7/30 (23.23%)	13/50 (26%)	0.790
**No**	23/30 (76.77%)	37/50 (74%)	
**Histological type**			
**IDC**	28/29 (96.55%)	26/30 (86.67%)	0.353
**Other**	1/29 (3.45%)	4/30 (13.33%)	
**SBR Grade**			
**Grade I**	2/26 (7.69%)	5/28 (17.86%)	0.027
**Grade II**	7/26 (26.92%)	15/28 (53.57%)	
**Grade III**	17/26 (65.38%)	8/28 (28.57%)	
**Mean tumor size (mm)**	27.15	36.15	0.201
**T stage**			
**T1-T2**	9/15 (60.00%)	9/14 (64.29%)	0.750
**T3**	1/15 (6.67%)	2/14 (14.29%)	
**T4**	5/15 (33.33%)	3/14 (21.42%)	
**Nodes involvement**			
**N+**	15/21 (71.43%)	14/26 (53.85%)	0.218
**N-**	6/21 (28.57%)	12/26 (46.15%)	
**Mean Ki-67 (%)**	38.79	34.95	0.598
**Ki-67 index status**			
**Ki-67 ≤14%**	1/19 (5%)	2/21 (9.52%)	1
**Ki-67>14%**	18/19 (95%)	19/21 (90.48%)	
**Molecular subtypes**			
**Luminal A**	1/23 (4.35%)	2/29 (6.90%)	0.926
**Luminal B**	12/23 (52.17%)	14/29 (48.28%)	
**Her2+**	1/23 (4.35%)	3/29 (10.34%)	
**TNBC**	9/23 (39.13%)	10/29 (34.48%)	
**ER receptor status**			
**RE+**	16/26 (61.54%)	23/37 (62.16%)	0.960
**RE-**	10/26 (385.46%)	14/37 (37.84%)	
**PR receptor status**			
**PR+**	14/26 (53.85%)	22/37 (59.46%)	0.658
**PR-**	12/26 (46.15%)	15/37 (40.54%)	
**HER2 receptor status**			
**HER2+**	2/25 (8.00%)	8/33 (24.24%)	0.163
**HER2-**	23/25 (92.00%)	25/33 (75.76%)	
**TNBC**			
**TNBC**	9/23 (39.13%)	10/29 (34.48%)	0.778
**Non-TNBC**	14/23 (60.87%)	19/29 (65.52%)	
**Metastatic status**			
**M0**	18/24 (75.00%)	22/32 (68.75%)	0.608
**M1**	6/24 (25.00%)	10/32 (31.25%)	

**Table 5:** Epidemiological and clinico-pathological characteristics of patients carrying *BRCA1* and *BRCA2* mutations.

Variables	*BRCA1+*N=17	*BRCA2+*N=14	*P* value
**Mean age at diagnosis (years)**	38.50	38.21	0.939
**Early age at onset (≤35 years)**			
**Yes**	7/16 (43.75%)	5/14 (35.71%)	0.654
**No**	9/16 (56.25%)	9/14 (64.29%)	
**Family history of breast cancer**			
**Yes**	16/17 (94.12%)	12/14 (85.71%)	0.576
**No**	1/17 (5.88%)	2/14 (14.29%)	
**Family history of ovarian cancer**			
**Yes**	8/17 (47.06%)	3/14 (21.43%)	0.258
**No**	9/17 (52.94%)	11/14 (78.57%)	
**Personal history of cancers**			
**Yes**	4/17 (23.53%)	1/14 (5.88%)	0.344
**No**	13/17 (76.47%)	13/14 (94.12%)	
**Consanguinity**			
**Yes**	2/17 (11.76%)	5/13 (38.46%)	0.190
**No**	15/17 (88.24%)	8/13(61.54%)	
**Histological type**			
**IDC**	15/15 (100%)	13/14 (92.86%)	0.483
**Other**	0/15	1/14 (7.14%)	
**SBR grade**			
**Grade I**	0/13	2/13 (15.38%)	0.673
**Grade II**	4/13 (30.77%)	3/13 (23.08%)	
**Grade III**	9/13 (69.23%)	8/13 (61.54%)	
**Mean tumor size (mm)**	25.36	29.33	0.555
**T stage**			
**T1-T2**	3/5 (60%)	6/10 (60%)	0.045
**T3**	1/5 (20%)	0/10	
**T4**	1/5 (20%)	4/10 (40%)	
**Nodes involvement**			
**N+**	6/10 (60%)	9/11 (81.82%)	0.361
**N-**	4/10 (40%)	2/11 (18.18%)	
**Mean Ki-67 (%)**	40.78	37.00	0.727
**Ki-67 index status**			
**Ki-67 ≤14%**	1/9 (11.11%)	0/10	0.474
**Ki-67>14%**	8/9 (88.89%)	10/10 (100%)	
**Molecular subtypes**			
**Luminal A**	1/13 (7.69%)	0/10	0.000078
**Luminal B**	2/13 (15.39%)	10/10 (100%)	
**Her2+**	1/13 (7.69%)	0/10	
**TNBC**	9/13 (69.23%)	0/10	
**ER receptor status**			
**RE+**	3/13 (23.08%)	13/13 (100%)	0.000056
**RE-**	10/13 (76.92%)	0/15	
**PR receptor status**			
**PR+**	2/13 (15.38%)	12/13 (92.31%)	0.000084
**PR-**	11/13 (84.62%)	1/13 (7.69%)	
**HER2 receptor status**			
**HER2+**	2/13 (15.38%)	0/12	0.480
**HER2-**	11/13 (84.62%)	12/12 (100%)	
**TNBC**			
**TNBC**	9/13 (69.23%)	0/10	0.002
**Non-TNBC**	4/13 (30.77%)	10/10 (100%)	
**Metastatic status**			
**M0**	8/11 (72.73%)	10/12 (83.33%)	0.640
**M1**	3/11 (27.27%)	2/12 (16.67%)	

### BRCA+ vs BRCA-

Family history of ovarian cancer was significantly associated with *BRCA* positive status (p=0.004). Regarding the mean age at diagnosis *BRCA* carriers seem to be younger than *BRCA-* patients (38.37 vs 43.14) (p= 0.049). However, no significant difference has been observed between both groups regarding family history of breast cancer, personal history of cancer and consanguinity. Similarly, no significant differences have been observed between the 2 groups in histological subtype, nodal involvement, tumor stage, hormonal receptors status, HER2 status, molecular subtypes, Ki-67 index, and metastases **(****Table 4****)**. Nevertheless, SBR grade III was found in 65.38% of patients with *BRCA1/2* mutations against a frequency of 28.57% among non-carriers, this difference appears to be statistically significant (p= 0.027).

### BRCA1 vs BRCA2

**Figure 4:**
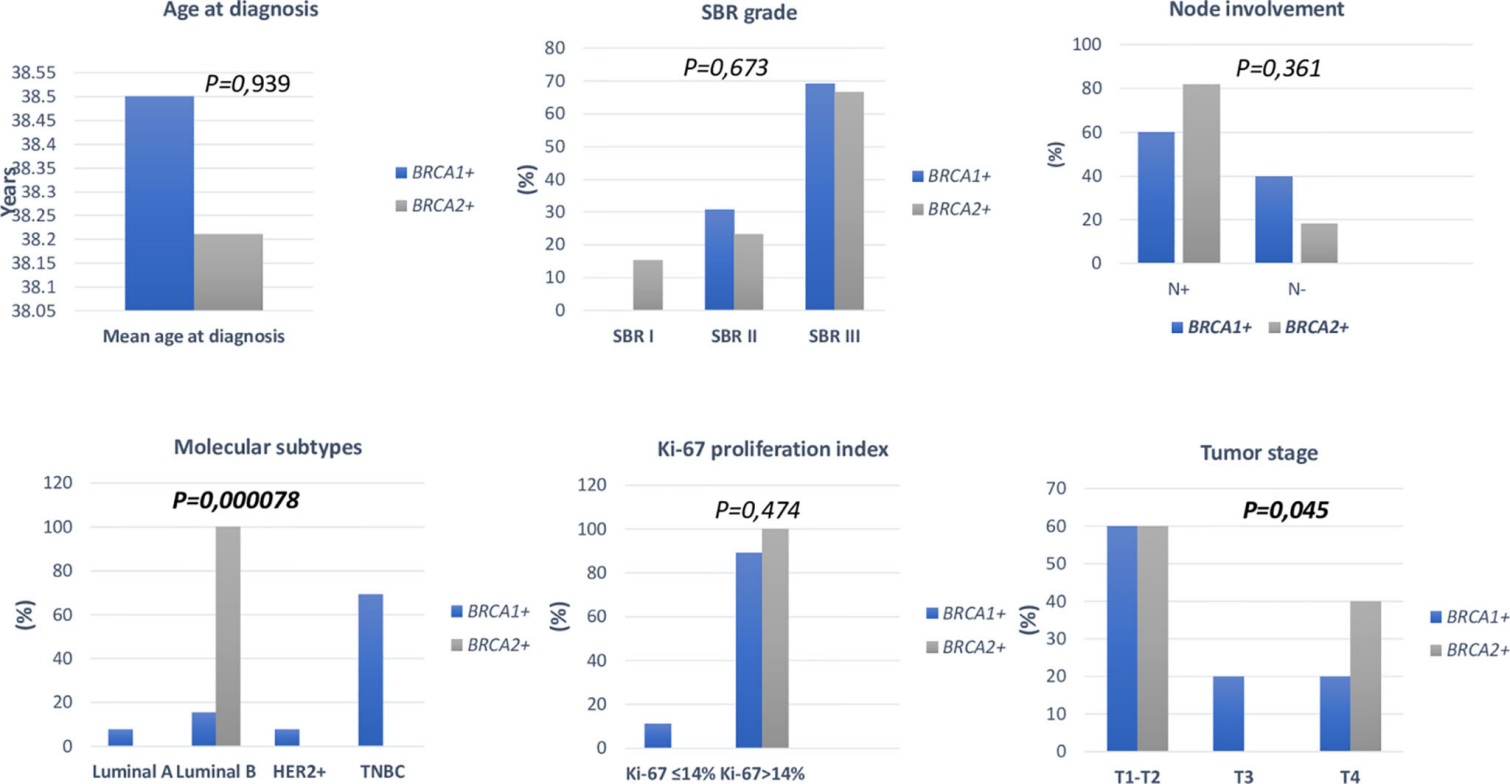
Distribution of clinico-pathological features of breast cancer in *BRCA1*+ and *BRCA2*+ patients.

**Figure 5:**
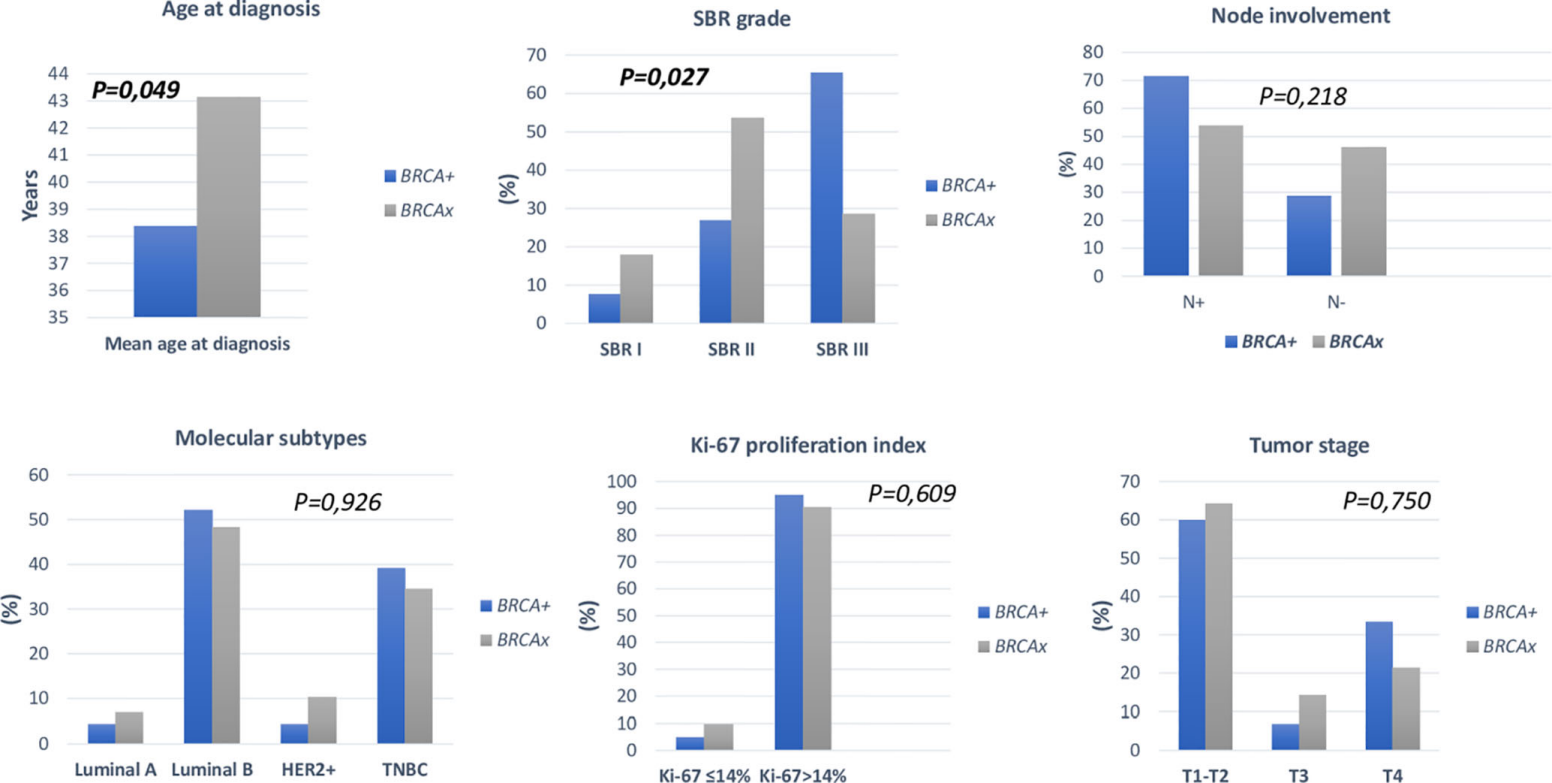
Distribution of clinico-pathological features of breast cancer in *BRCA*+ and *BRCAx* patients.

Association between clinico-pathological features and *BRCA* status (*BRCA1+, BRCA2+* and *BRCAx*) was shown in **Figures 4**, **5**. Our results showed that there were no significant differences between *BRCA1* and *BRCA2* mutated groups regarding the mean age at diagnosis, the family history of personal cancers, of breast cancer and ovarian cancer (**Table 5**).

Pathology showed that the infiltrating ductal carcinoma was the most common histological type in both groups (100% and 92.86%). HER2 status, lymph node involvement, SBR grade, tumor size, Ki-67 index and metastatic status showed no statistically significant difference between both studied groups. However, *BRCA1* carriers were more likely to have triple negative breast cancer (p=0.002) and *BRCA2* carriers were more likely to have luminal B breast cancer tumors (p=0.000078). In addition, positive estrogen receptor (ER) status and positive progesterone receptor (PR) status studied separately were both associated with *BRCA2* mutated tumors (p=0.000056 and p=0.000084), respectively.

### Follow Up of *BRCA1* and *BRCA2* Carriers

Among *BRCA* carriers, contralateral breast cancer and ovarian cancer co-occurrence were observed respectively in 22.58% and 16.12% of cases. One patient diagnosed with early onset breast cancer has undergone a contralateral prophylactic mastectomy and is currently under regular surveillance. Both contralateral breast cancer and ovarian cancer occurrence were more frequent in *BRCA1* than *BRCA2* carriers. Also, 22.58% of the carriers have developed distant metastases and 5 cases died due to disease progression.

## Discussion

Detection of mutations in hereditary breast and ovarian cancer related *BRCA1* and *BRCA2* genes is an effective method of cancer prevention, early detection, and treatment. Mutations in the highly penetrant *BRCA* genes explain around a quarter of these cases (27). The frequency of germline mutations identified on both genes varies depending on the geographic and ethnic distributions. In some populations, a wide spectrum of different mutations is present, whereas in other groups specific recurrent *BRCA* mutations have been reported that may be due to the founder mutation effect (28–32).

Our previous studies, investigating breast cancer loci and Nucleotide Excision Repair pathway, have shown that the Tunisian population is an admixed and intermediate population between Sub-Saharan Africans and Europeans (33, 34). This genetic diversity reflects the inter-ethnic variability in the frequency distribution of the studied polymorphisms. Indeed, allele frequencies of several variants were found to be statistically different between Tunisian and other populations including rs2046210 and rs941764 that site in breast cancer susceptibility loci (33). These findings are in favor of the genetic heterogeneity to breast cancer predisposition in the Tunisian population. So far, only 18 deleterious *BRCA* mutations have been reported. In the current study, 16 *BRCA* mutations, including 11 novel variations, have been identified in a cohort of 354 Tunisian breast and ovarian cancer patients. For breast cancer cases, high fractions of young patients (31.94%), cases with family history of breast cancer (35.24%), Triple negative breast cancer (24.31%) and high tumor grade (47.41%) have been observed. As reported in previous studies, the high fractions of early onset, triple negative cases and also the presence of family history of breast cancer may be associated with germline *BRCA* mutations (35, 36). Indeed, it is now well documented that breast cancer patients in North Africa are almost 10 years younger than patients from western countries (37). In Tunisia, around 11% of breast cancer cases are under 35 years old (38). In fact, at a young age, the human organism usually functions as well as it ever will. However, interactions between some genetic and environmental factors (GxE) may cause a physiological decline of some organism systems leading to early disease presentation. Therefore, the influence of specific genetic background, differences in variant penetrance and frequency between populations along with environmental factors may explain this early onset of the disease. Large cohorts of young breast cancer patients should be studied to elucidate these GxE factors.

For ovarian cancer cases, the mean age at diagnosis was 52.62 years and the majority presented with serous ovarian carcinoma. Previous studies have shown that among all patients diagnosed with serous ovarian carcinoma, which is the most common subtype, over 15% will have germline *BRCA* mutations (39).

Among the 16 distinct deleterious mutations that have been observed c.19_47del, c.668dupA, c.915T>A, c.1612C>T, c.2418dupA, c.2433delC, c.3049G>T and c.5030_5033delCTAA in *BRCA1* and c.-227-?_7805+? (Del exons 1-16), c.249delG, c.632-1G>A, c.1389_1390delAG in *BRCA2*, are reported for the first time in the Tunisian population. We have also identified an inframe deletion reported to have a conflicting interpretation of pathogenicity effect in early onset bilateral breast cancer patient *BRCA1*_c.5017_5019delCAC. This variation has been described in multiple breast and ovarian cancer cases, with some families showing incomplete co-segregation of the variation (40–42).

Among *BRCA* mutations identified in breast cancer patients *BRCA1_c*.211dupA, *BRCA1*_c.5266dupC,*BRCA2*_c.-227-?_7805+?,*BRCA2*_1310_1313delAAGA, *BRCA2*_c.1389_1390delAG and *BRCA2*_c.7654dupA occurred in BCCRs that are considered to be associated with an increased likelihood of breast cancer compared to ovarian cancer. Considering *BRCA* mutations identified in ovarian cancer patients *BRCA1*_c.1612C>T and *BRCA1*_3049G>T arose in OCCRs. Other mutations, namely c.19_47del, c.668dupA, c.915T>A, c.2418dupA, c.2433delC, c.5030_5033delCTAA in *BRCA1* and c.249delG, c.632-1G>A in *BRCA2* do not overlap with previously reported breast or ovarian cancer cluster regions. This could be explained by ethnic differences in *BRCA* mutation spectrum or it may indicate shared cluster regions for both breast and ovarian cancer.

In the *BRCA1* gene, the c.19_47del mutation was identified in one breast cancer patient. This mutation was previously described only in the Algerian population (43). The c.2433delC mutation was described in Korean breast and ovarian patients (44, 45), and in Mexican patients (46, 47). The pathogenic c.1612C>T mutation was identified in 4 breast and ovarian cancer patients. This mutation has been identified in Brazilian population (48), in ovarian cancer patients from Israeli population (49) and in Macedonian population (50). We also detected the c.668dupA mutation in one patient. This latter has not been reported in previous studies neither in Tunisia nor in other populations. Nevertheless, it is already listed and classified as pathogenic in ClinVar and predicted to result in the substitution of Alanine to Glycine (p.Ala224Glyfs) which leads to BRCA1 protein truncation. Another new mutation was identified in *BRCA1* gene, c.2418dupA, that was reported by our group for the first time in the Tunisian population and was not reported previously in other populations (51). c.3049G>T has been identified in one ovarian cancer patient. This mutation has been reported in Thai patients with non-mucinous epithelial ovarian cancer (52). The c.5030_5033delCTAA mutation was identified among one patient with breast and ovarian cancers and it is reported in Brazilian population (48). The c.915T>A mutation is novel and not described in public databases.

In addition to the identification of rare and novel *BRCA1* mutations, other mutations seem to be recurrent and/or were described in previous Tunisian reports. The c.211dupA mutation was shared by 6 patients belonging to the same geographical origin. This mutation has so far been reported only in hereditary breast/ovarian cancer families of Tunisian origin, particularly in the North-East region, suggesting a founder effect. In order to unravel the genetic specificities of this mutation and to trace its origin a haplotype analysis has been conducted by our group on the North Eastern region (51). Results have determined the founder haplotype segregating with this mutation and have revealed that it arose in the period of colonization approximately 130 years ago.

The c.5266dupC mutation has been identified among two families. This mutation was previously described in 8 Tunisian breast cancer families (11, 16, 17, 20). It was originally described as an Ashkenazi founder mutation. Haplotype analysis has shown that this mutation arose approximately 1800 years ago in Northern Europe (53). Then, it has been reported in several other populations such as, Italian, Russian Slovenian and Greek (54).

Interestingly for *BRCA2* gene, 6 breast cancer patients were double heterozygous carrying the two deleterious mutations c.632-1G>A and c.1310_1313delAAGA, and 4 other unrelated patients carried only the c.1310_1313delAAGA mutation including one male breast cancer (MBC). c.632-1G>A mutation appears to be rare in other populations since it was only reported in one patient with prostate cancer in the UK (55). However, c.1310_1313delAAGA seems to be a founder mutation in Maghrebin countries (16, 17, 56, 57). It has been also identified in patients with Lebanese (58), European (59–62), African (63), Asian (64) and Latino ancestry (65) as well as in Caribbean cohorts (66, 67). These results show the genetic heterogeneity of breast and ovarian cancers in Tunisian patients and the admixed origins of *BRCA* mutations in Tunisia.

In addition, 5 male breast cancer cases were investigated among which 2 carried *BRCA2* mutations (c.1310_1313delAAGA and c.1389_1390delAG). Male breast cancer is a rare disease accounting for less than 1% of all breast cancer cases and it was previously shown that nearly 90% of MBC arising in *BRCA* mutation carriers are found to harbor a *BRCA2* mutation (68). Unfortunately, being a man with “a women’s disease” makes MBC a disease surrounded by social taboo and lack of awareness especially in underdeveloped countries. Indeed, the treatment of MBC has been extrapolated from the knowledge of female breast cancer, despite the multiple differences in the pathogenesis, biology and genetics of these two disease entities. These evidence make MBC a gender issue that requires more attention from the scientific community.

The introduction of the c.1310_1313delAAGA mutation, that have been encountered in diverse populations, in the Tunisian population could be explained by the immigration of Andalusians in Tunisia which has been intensified after the fall of Granada in 1492 and lasted for two centuries before the total expulsion of all Andalusian Moriscos from the Iberian Peninsula in 1610. The diverse geographical distribution of this mutation may further suggest independent origins as shown for the 4184del4 *BRCA1* mutation reported to have at least three independent origins in the study of Neuhausen et al. (69). The c.7654dupA *BRCA2* gene mutation which was identified in a unique family with a strong family history of breast and ovarian cancer is reported previously and exclusively in Algerian population (70) and could be therefore specific to North African countries.

Through this report and despite the identification of novel mutations in Tunisian population, it is clear that the genetic susceptibility to breast cancer is explained in a vast majority of cases by recurrent mutations. Indeed, more than 44.44% of carriers harbor *BRCA1*-c.211dupA or *BRCA2*-1310_1313deAAGA mutations which highlights the importance of screening these mutations in the treatment workflow of cases with early onset or strong family history of breast cancer. In fact, identifying germline *BRCA1* and *BRCA2* pathogenic mutations is a crucial component in the medical management of affected patients. Regular surveillance and/or prophylactic mastectomy of the second breast or prophylactic salpingo oophorectomies, which have been shown to reduce the risk of developing cancer, are recommended to these carriers. Moreover, relatives who test positive for a germline *BRCA* pathogenic mutation may take appropriate action to prevent cancer or have cancer diagnosed as early as possible for better treatment options (59).

In addition, mutations in the *BRCA* genes and their associations with clinico-pathological features were reported in several studies (71–74). However, in Tunisia this aspect was not previously investigated. This point was raised in the present study and our results showed that patients with *BRCA1* and *BRCA2* mutations were similar with regard to several epidemiological and clinico-pathological parameters. Nevertheless, *BRCA1* carriers were more likely to be triple negative breast cancer compared to *BRCA2* carriers (p=0.002) and *BRCA2* carriers were more likely to be luminal B breast cancer tumors (p=0.000078). Consistent with our findings, various previous studies reported that there is a much higher rate of TNBC among *BRCA1* mutation carriers (75, 76) and *BRCA2*-related breast cancer is often luminal (77). Additionally, positive ER was significantly associated with *BRCA2+* tumors (p=0.000056). PR status was significantly different between *BRCA1* and *BRCA2* mutation carriers; *BRCA2* carriers are more likely to develop progesterone receptor (PR) positive tumors and PR-negative breast cancer are associated with *BRCA1* mutation carriers (p=0.000084). It was reported that the ER positivity was predominantly seen in *BRCA2* mutation carriers, which is consistent with our findings (71, 78). Furthermore, a previous report has found that *BRCA2*-associated cancers are mainly PR positive (79). Other studies have raised some pathological differences between *BRCA1/2* mutation carriers and *BRCAx* patients. In our study, *BRCA* carriers seem to be younger than *BRCA-negative* patients (p=0.049). Furthermore, patients with a positive family history of ovarian cancer are more likely to be *BRCA* positive (p=0.004). We also observed a significant predominance of SBR grade III tumors among *BRCA1/2* mutations carriers (p= 0.027). These findings are in line with previous literature  (35, 80, 81).

Furthermore, we have assessed disease outcomes in *BRCA* carriers, and we have observed a relatively high proportion of contralateral breast cancer and ovarian cancer occurrence that were more frequently observed in *BRCA1* carriers. Previous reports have demonstrated that women carrying a pathogenic mutation in the *BRCA1* or *BRCA2* genes have an increased risk of developing a second primary cancer in the contralateral breast. The cumulative risk 20 years after breast cancer diagnosis was estimated to be 40% for *BRCA1* carriers and about 26% for *BRCA2* carriers (82). In accordance with our findings, it was shown also that the occurrence of both breast and ovarian cancer in a woman is associated with a high likelihood of a germline *BRCA1* mutation (83).

Besides the *BRCA* genetic mutations that have been identified in our study, mutations on other high to moderate breast cancer genes such as *TP53*, *ATM, BLM* and *CHEK2* have been also identified for the first time in North African populations (data not shown). All these findings reflect the genetic heterogeneity of cancer predisposition in Tunisia and highlights the importance of the use of NGS to identify clinically actionable genetic variants that have a crucial role in disease management. Therefore, technological advances in terms of array and DNA sequencing technologies made the route towards the examination of genetic risk largely clear. However, practical challenges related to marked population-specific differences still exist. In this context, Manolio and colleagues conveniently classified *LRRK2* as a high penetrant gene associated with Parkinson disease (84) with G2019S mutation being the main cause of Parkinson familial cases. Recently the international LRRK2 consortium reported a worldwide frequency of 1% of *LRRK2* G2019S, 30–40% in Arab patients from North Africa and 10–30% in Ashkenazi Jews, but is very rare in Asians (85, 86). As a variant´s frequency has a direct impact on its penetrance, this example shows the ethnic-dependent penetrance of some important variants involved in complex diseases and the role of consanguinity and endogamy in shaping the genetic susceptibility to these diseases. Therefore, the same reflection can be applied on high and low penetrant breast cancer variants in order to review their penetrance in underrepresented populations such as North Africans. A disproportionate distribution of the identified mutations is observed between the Northern and Southern regions of Tunisia (**Figure 2**), with the vast majority found in the North. This can be explained by a selection bias because most of the recruited participants come from Northern governorates, but it can also be explained by the very high consanguinity rates in the South that reaches 98% in some cities and that may have an impact on *BRCA* mutations frequency and prevalence.

Additional limitations of our study have been observed. Indeed, to our best knowledge, this work represents the largest BRCA1/2 study in Africa. However, we believe that the sample size is still small and larger cohorts are needed to trace a clear and complete BRCA1/2 mutational spectrum in Tunisia. In addition, because of the limited resources dedicated to this work, we were not able to perform a complete sequencing of both genes for the whole cohort. Therefore, the frequency and prevalence of the identified mutations need to be assessed in larger studies. Clearly, the prevalence assessment of *BRCA1* and *BRCA2* mutations also rely on the quality of both cohort selection criteria and mutation ascertainment methods. The identification of novel BRCA mutations and the assessment of their penetrance in a specific population will help to implement more affordable and cost effective targeted genetic testing strategies.

Finally, up until now, most data on *BRCA1*/2 mutations associated with high risk for hereditary breast and ovarian cancer do not cover the North African populations. Accordingly, the novel mutations identified in this study will help to improve knowledge on the genetic component of hereditary breast and ovarian cancer in the North African region and will lead to a better clinical management of cancer patients. In addition, we are aiming to share genetic and phenotypic data with larger multi-ethnic Consortia of *BRCA1/2* mutation carriers such as the Consortium of Investigators of Modifiers of BRCA1/2 (CIMBA) (87). This will make our findings more broadly useful and will give us a global overview of the similarities and differences that the Tunisian population has compared to other ethnicities.

## Conclusion

In conclusion we have identified 16 distinct *BRCA* mutations in breast and ovarian cancer patients including 11 novel mutations in the Tunisian population. The recognition of the *BRCA* mutational spectrum and its geographical distribution in Tunisia is of keen interest for the scientific and medical communities as it helps to develop precise risk assessment tools, accurate genetic testing, cost-effective approaches for prevention and early detection of the disease as well as personalized treatments of *BRCA* related cancers for both affected and unaffected cancer cases.

## Data Availability Statement

The minimal dataset that would be necessary to interpret, replicate and build upon the findings reported in this study are included in this article and in its supplementary files. All identified mutations with their related details have been shared in the public database ClinVar under the following link “https://www.ncbi.nlm.nih.gov/clinvar/submitters/507986/”. Any additional datasets used and/or analyzed during the current study are available from the corresponding author on reasonable request.

## Ethics Statement

The studies involving human participants were reviewed and approved by The Biomedical Ethics Committee of Institut Pasteur de Tunis (2017/16/E/Hôpital A-M). The patients/participants provided their written informed consent to participate in this study. Written informed consent was obtained from participants for the publication of any potentially identifiable images or data included in this article.

## Author Contributions

YH prepared the study concept and design, supervised the study, did data analysis, data interpretation, drafted, and critically revised the manuscript. NMi and MB did the experiments, participated in participant recruitment, and participated in drafting and reviewing the manuscript. NMe contributed to clinical data analysis and reviewed the manuscript. SN contributed to participants recruitment and reviewed the manuscript. MR and OM contributed to data collection and reviewed the manuscript. HanB, YB, HR, OJ, ND, AZ, JA, HEB, SL and JBH contributed to the clinical investigation and recruitment of patients. AH, KR, FB, RM, SBA and HamB critically revised the clinicopathological section and the whole manuscript. SB and SA contributed to the study concept, design and supervision and critically revised the manuscript. All authors contributed to the article and approved the submitted version.

## Funding

This work was supported by the Tunisian Ministry of Higher Education and Scientific Research (LR16IPT05) and the Tunisian Ministry of Public Health (PEC-4-TUN). MB and MBR are recipient of a MOBIDOC fellowship funded by the EU through the EMORI and PASRI programs managed by the ANPR.

## Conflict of Interest

The authors declare that the research was conducted in the absence of any commercial or financial relationships that could be construed as a potential conflict of interest.

## Publisher’s Note

All claims expressed in this article are solely those of the authors and do not necessarily represent those of their affiliated organizations, or those of the publisher, the editors and the reviewers. Any product that may be evaluated in this article, or claim that may be made by its manufacturer, is not guaranteed or endorsed by the publisher.

## Abbreviations

BCCRs, Breast Cancer Cluster Regions; ER, Estrogen receptor; gDNA, Genomic DNA; GxE, Genetic and Environmental factors; IDC, Invasive Ductal Carcinoma; LMICs, Low- and middle-income countries; OCCRs, Ovarian cancer cluster regions; PR, Progesterone receptor; SBR, Scarff-Bloom-Richardson; TNBC, Triple Negative Breast Cancer; VUS, Variants of Unknown Significance; WES, Whole Exome Sequencing.
