# Supplementary material for: Identification of Eleven Novel BRCA Mutations in Tunisia: Impact on the Clinical Management of BRCA Related Cancers
Source: Front Oncol. 2021 Aug 20;11:674965. doi: 10.3389/fonc.2021.674965 (PMC8417726; doi:10.3389/fonc.2021.674965)
Supplement: Supplementary file 1 [file Table_1.docx]

**Table S1. Polymorphisms and Variant of unknown significance identified in breast and ovarian cancer cases.**

| Gene | Localisation | Variant ID | Nucleotide change | Amino Acide Change | gnomAD_genome | ClinVar |
| --- | --- | --- | --- | --- | --- | --- |
| ***BRCA1*** | Intronic | rs375078966 | c.5468-123_5468-121del | . | 0.0060 | . |
|  | Exonic | rs4986852 | c.3119G>A | S1040N | 0.0112 | Benign |
|  | Intronic | rs799923 | c.442-34C>T | . | 0.1545 | Benign |
|  | Intronic | rs3092994 | c.5152+66G>A | . | 0.3135 | Benign |
|  | Intronic | rs8176257 | c.5075-237C>A | . | 0.2432 | Benign |
|  | Intronic | rs8176235 | c.5074+65G>T | . | 0.2497 | Benign |
|  | Intronic | rs8176234 | c.4987-68A>G | . | 0.3143 | Benign |
|  | Intronic | rs8176233 | c.4987-92A>G | . | 0.3123 | Benign |
|  | Intronic | rs3092987 | c.4986+222A>G | . | 0.3018 | Benign |
|  | Exonic | rs1799966 | c.4837A>G | S1613G | 0.3179 | Benign |
|  | Intronic | rs273900734 | c.4485-63C>G | . | 0.3160 | Benign |
|  | Intronic | rs8176194 | c.4358-2590T>G | . | 0.3008 | Benign |
|  | Intronic | rs8176193 | c.4358-2885G>A | . | 0.3166 | Benign |
|  | Exonic | rs1060915 | c.4308T>C | S1436S | 0.3011 | Benign |
|  | Intronic | rs799916 | c.4097-141A>C | . | 0.4502 | Benign |
|  | Exonic | rs16942 | c.3548A>G | K1183R | 0.3150 | Benign |
|  | Exonic | rs16941 | c.3113A>G | E1038G | 0.3004 | Benign |
|  | Exonic | rs799917 | c.2612C>T | P871L | 0.4855 | Benign |
|  | Exonic | rs16940 | c.2311T>C | L771L | 0.2998 | Benign |
|  | Exonic | rs1799949 | c.2082C>T | S694S | 0.3160 | Benign |
|  | Intronic | rs8176144 | c.407-58del | . | 0.3001 | Benign |
|  | Intronic | rs8176140 | c.547+146A>T | . | 0.3152 | Benign |
|  | Intronic | rs799905 | c.-20+101C>G | . | 0.4941 | Benign |
|  | Intronic | rs8176145 | c.593+167T>C | . | 0.3156 | Benign |
|  | Intronic | rs72434991 | c.441+63_441+64del | . | 0.5428 | . |
|  | Intronic | rs373413425 | c.441+36_441+49del | . | 0.2780 | Benign |
|  | Intronic | rs8176305 | c.5333-153A>G | . | 0.0519 | Benign |
|  | Exonic | rs4986850 | c.2077G>A | D693N | 0.0540 | Benign |
|  | Intronic | rs3765640 | c.-19-115T>C | . | 0.3166 | Benign |
|  | Exonic | rs1799965 | c.591C>T | C197C | 0.0017 | Benign |
|  | Intronic | rs8176316 | c.5468-10C>A | . | 0.0039 | Benign |
|  | Exonic | rs1799950 | c.1067A>G | Q356R | 0.0515 | Benign |
|  | Intronic | rs8176306 | c.5333-134C>A | . | 0.0019 | Benign |
|  | UTR3 | rs8176318 | c.*421G>T | . | 0.2974 | Benign |
|  | Intronic | rs8176297 | c.5278-191A>T | . | 0.3133 | Benign |
|  | Exonic | rs1799967 | c.4956G>A | M1652I | 0.0196 | Benign |
|  | Intronic | rs3737559 | c.4357+117G>A | . | 0.0802 | Benign |
|  | Exonic | rs80358343 | c.5017_5019delCAC | H1673del | . | Conflicting interpretations of pathogenicity​ :  Likely pathogenic(3);Uncertain significance(4) |
|  | Exonic | rs56012641 | c.1648A>C | N550H | 0.00008818 | Benign |
|  | Exonic | rs55906931 | c.1456T>C | F486L | 0.00008819 | Benign |
|  | Exonic | rs56187033 | c.536A>G | Y179C | 0.0002229 | Benign |
|  | Intronic | rs772583635 | c.547+7G>A | . | 0.000003977 | Likely benign |
|  | Exonic | rs80356860 | c.5117G>C | G1706A | 0.0001274 | Benign |
|  | Exonic | rs1800709 | c.2521C>T | R841Trp | 0.001083 | Benign |
|  | Exonic | rs1800704 | c.3024G>A | Met1008I | 0.0001911 | Benign |
|  | Exonic | rs1800740 | c.2733A>G | G911G | 0.0001911 | Likely Benign |
|  | Exonic | rs1800063 | c.981A>G | P798S | 0.0001910 | Benign |
|  | Intronic | rs8176128 | c.212+23T>A | . | rs8176128 | Benign |
|  | Intronic | rs921962743 | c.441+51del | . | 0.001143 | . |
| ***BRCA2*** | Intronic | rs206073 | c.793+98G>A | . | 0.9797 | Benign |
|  | Exonic | rs144848 | c.1114A>C | N372H | 0.2218 | Benign |
|  | Exonic | rs206075 | c.4563A>G | L1521L | 0.9799 | Benign |
|  | Exonic | rs206076 | c.6513G>C | V2171V | 0.9798 | Benign |
|  | Intronic | rs206080 | c.6938-120T>C | . | 0.9798 | Benign |
|  | Exonic | rs169547 | c.7397T>C | V2466A | 0.9819 | Benign |
|  | Exonic | rs4987047 | c.8830A>T | I2944F | 0.0112 | Benign |
|  | Intronic | rs206096 | c.7617+190G>A | . | 0.9985 | Benign |
|  | Exonic | rs543304 | c.3807T>C | V1269V | 0.1816 | Benign |
|  | Intronic | rs2126042 | c.681+56C>T | . | 0.2161 | Benign |
|  | Intronic | rs2320236 | c.1910-74T>C | . | 0.2053 | Benign |
|  | Intronic | rs9534262 | c.7806-14T>C | . | 0.5470 | Benign |
|  | Intronic | rs4942486 | c.8755-66T>C | . | 0.5258 | Benign |
|  | Exonic | rs768907899 | c.5688A>G | A1896A | 9.691e-05 | Likely benign |
|  | Intronic | rs11571744 | c.8487+47C>T | . | 0.0133 | Benign |
|  | UTR5 | rs1799943 | c.-26G>A | . | 0.2208 | Benign |
|  | Intronic | rs11571610 | c.425+67A>C | . | 0.0307 | Benign |
|  | Exonic | rs766173 | c.865A>C | N289H | 0.0307 | Benign |
|  | Exonic | rs1801439 | c.1365A>G | S455S | 0.0306 | Benign |
|  | Intronic | rs11571651 | c.1910-51G>T | . | 0.0305 | Benign |
|  | Exonic | rs1801499 | c.2229T>C | H743H | 0.0308 | Benign |
|  | Exonic | rs1799944 | c.2971A>G | N991D | 0.0375 | Benign |
|  | Exonic | rs1801406 | c.3396A>G | K1132K | 0.2984 | Benign |
|  | Intronic | rs11571661 | c.6841+80_6841+83del | . | 0.2932 | Benign |
|  | Exonic | rs1799955 | c.7242A>G | S2414S | 0.2299 | Benign |
|  | Intronic | rs11147489 | c.7435+53C>T | . | 0.0303 | Benign |
|  | Intronic | rs3764791 | c.8754+183A>C | . | 0.2107 | Benign |
|  | Intronic | rs3764792 | c.8754+187C>T | . | 0.2248 | Benign |
|  | Exonic | rs11571769 | c.8851G>A | A2951T | 0.0037 | Benign |
|  | Intronic | rs144549870 | c.1909+92_1909+96del | . | 0.0130 | Benign |
|  | Intronic | rs11571818 | c.9257-16T>C | . | 0.0054 | Benign |
|  | Exonic | rs11571833 | c.9976A>T | K3326X | 0.0053 | Benign |
|  | Exonic | rs1801426 | c.10234A>G | I3412V | 0.0372 | Benign |
|  | Intronic | rs11571574 | c.67+62T>G | . | 0.0025 | Benign/Likely benign |
|  | UTR3 | rs15869 | c.*105A>C | . | 0.1563 | Benign |
|  | Intronic | rs191765802 | c.316+183T>G | . | 0.0004 | . |
|  | Intronic | rs11571680 | c.7007+116TTTATAAAA | . | 0.0304 | Benign |
|  | Intronic | rs950067722 | c.8488-66G>A | . | . | . |
|  | Exonic | rs11571746 | c.8503T>C | S2835P | 0.0020 | Benign |
|  | UTR5 | rs206118 | c.-806A>G | . | 0.1499 | Benign |
|  | Intronic | rs9595456 | c.9257-83G>A | . | 0.0415 | Benign |
|  | Exonic | rs397507308 | c.3422C>T | T1141I | 0.000003984 | Uncertain significance |
|  | Exonic | rs1057524039 | c.7296A>G | R2432R | . | Likely benign |
|  | Exonic | rs9590940 | c.8460A>C | V2820V | 0.03553 | Benign |
|  | Exonic | rs1555283421 | c.3837T>C | N1279N | . | Likely benign |
|  | Exonic | rs41293521 | c.9292T>C | Y3098H | 0.0001274 | Benign |
|  | Exonic | rs11571642 | c.1788T>C | D596D | 0.007462 | Benign |
|  | Exonic | rs80358755 | c.5312G>A | G1771D | 0.00008826 | Benign |
|  | Exonic | rs28897724 | c.4068G>A | L1356L | 0.002453 | Benign |
|  | Intronic | rs276174878 | c.68-7del | . | 6.638e-05 | Conflicting interpretations of pathogenicity​  Benign(2);Uncertain significance(2) |
|  | Intronic | rs276174816 | c.1909+22del | . | 0.0026 | Conflicting interpretations of pathogenicity​  Benign(6);Likely benign(1);Uncertain significance(1) |
|  | Intronic | rs751977993 | c.793+63del | . | 0.0027 | Benign |
